# Richmond Academy of Medicine and Surgery

**Published:** 1897-06

**Authors:** Mark W. Peyser

**Affiliations:** Secretary and Reporter


### Society Reports.

### RICHMOND ACADEMY OF MEDICINE AND SURGERY.

Regular meeting held May 11, 1897. Dr. J. N. UPSHUR, President, in the chair. Dr. MARK W. PEYSER, Secretary and Reporter.

Dr. W. S. BEAZLEY read a paper on

### SOME OF THE EVILS THAT THREATEN THE MEDICAL PROFESSION.

### REPORT OF CASES.

Dr. GEORGE ROSS reported the following cases:

*Malignant Diseases in Child's Mouth.*—Boy, age eight years. Father, mother and uncle died of consumption. Aunt has it. Grandmother died of apoplexy. An abscess in the lower jaw due to a decayed molar, was opened several times, and now, in the cavity, there has appeared a foreign growth, about the size of a guinea egg, and apparently of a malignant character. He reported the case because of the rarity of malignant growths in childhood.

*Myelitis and Paraplegia. Partial Recovery. Then Right and, Later, Left Hemiplegia. Paralysis of Diaphragm and Death.*—Male, white, aged about fifty-eight years; wore low quartered shoes the whole of one rainy day, and the afternoon, without changing them, put on rubber shoes. The next morning he complained of chilliness and malaise. Walking became more and more difficult until the fifth day, when it was an impossibility to walk. A diagnosis of acute myelitis was made with paraplegia—paralysis of the bladder and rectum, etc. He removed South, remaining an ataxic, then went North, and consulted various authorities, Dr. Seguin among others.

About twelve days ago, while in the city again, he became absolutely paralyzed on the right side, and was taken to the Virginia Hospital. Six days ago there was a second paralytic attack involving the left side and the diaphragm. In twenty-four hours articulation became impossible, and he died.

The patient was a student and literary man, with no syphilitic or gouty history, and of regular habits.

The President said Dr. Ross's case was very interesting, especially because of the absence of gouty or syphilitic history. He thought there must have been some condition, back of the myelitis, tending to produce tissue change. The patient might have *inherited* gout, and along with it slow changes in the vascular system, as deficiency of the elasticity of the vessels, or a change due to the cold going to the brain and slowly acting upon the nervous tissue.

He reported the following case:

*Suppurative Salpingitis. Recovery under Medical Treatment.*— Woman, age twenty-three years; unmarried; seen January 28, at which time she had been confined to bed for five months. She complained of pain at all times; and between 12 M. and 3 P. M. of violent attacks in the hypogastrium.

There was also dysmenorrhœa. Digital examination revealed the fact that she was not a virgin. The uterus was firmly fixed in the pelvis, and its body could not be outlined. In the cul-de-sac, a mass found as large as an orange, and boggy to the touch. A dirty discharge came from the cervix. There was occasional difficulty with micturition. After tonic treatment and the use of douches at home, she was taken to the Old Dominion Hospital for operation, and Dr. J. W. Long was called in consultation. They agreed that the case was suppurative salpingitis, due in all probability to gonorrhœa. Twice daily douches of normal saline solution, as hot as could be borne, were given, followed by the introduction of a tampon anointed with 25 per cent. ichthyol unguent.

At the end of six weeks the effusion had entirely disappeared, and the only evidence of pelvic trouble was the dirty discharge.

Menstruation at this time was still irregular, but not attended by pain. Ten days ago the uterus was dilated and curetted (there being hardly any suppuration), and packed with iodoform gauze. In three days she returned, and very little discharge was seen. He now uses a syringe (capacity, one drachm) to inject pure peroxide of hydrogen, and then swabs out the uterus with ichthyol. Absolute recovery is evidenced, and that without operation. Tonic treatment consisted in the administration, three times daily, of a pill of manganese, iron, and nux vomica.

Dr. JACOB MICHAUX reported in detail a case of

*Abscess of the Kidney*, originating from a neglected case of inflammaton of the bladder. Urine secreted, although about one-half of kidney was taken up in an abscess cavity. [Dr. Michaux has the case still under treatment, and as the other kidney is becoming involved, he will reserve a full report of the case until the next issue of this journal.]

